# Joint Function and Movement Variability During Daily Living Activities Performed Throughout the Home Setting: A Digital Twin Modeling Study

**DOI:** 10.3390/s25247409

**Published:** 2025-12-05

**Authors:** Zhou Fang, Mohammad Yavari, Yiqun Chen, Davood Shojaei, Peter Vee Sin Lee, Abbas Rajabifard, David Ackland

**Affiliations:** 1 Department of Biomedical Engineering, University of Melbourne, Melbourne, VIC 3010, Australiamohammad.yavari@student.unimelb.edu.au (M.Y.); pvlee@unimelb.edu.au (P.V.S.L.); 2 Department of Infrastructure Engineering, University of Melbourne, Melbourne, VIC 3010, Australia

**Keywords:** mobility, wearable sensor, IMU, machine learning, remote monitoring, joint kinematics

## Abstract

**Highlights:**

**What are the main findings?**

- A digital twin was developed to support real-time mobility and joint motion measurement and monitoring in the home setting.
- Activities of daily living, and the way they are executed, vary throughout the home setting, even for the same task.

**What are the implications of the main findings?**

- The findings have implications for home interior design and layout to improve mobility and reduce the risk of falls.
- The framework presented may be useful for real-time monitoring of movement in the home setting, as well as telemedicine and telerehabilitation.

**Abstract:**

Human mobility is commonly assessed in the laboratory environment, but accurate and robust joint motion measurement and task classification in the home setting are rarely undertaken. This study aimed to develop a digital twin model of a home to measure, visualize, and classify joint motion during activities of daily living. A fully furnished single-bedroom apartment was digitally reconstructed using 3D photogrammetry. Ten healthy adults performed 19 activities of daily living over a 2 h period throughout the apartment. Each participant’s upper and lower limb joint motion was measured using inertial measurement units, and body spatial location was measured using an ultra-wide band sensor, registered to the digital home model. Supervised machine learning classified tasks with a mean 82.3% accuracy. Hair combing involved the highest range of shoulder elevation (124.2 ± 21.2°), while sit-to-stand exhibited both the largest hip flexion (75.7 ± 10.3°) and knee flexion (91.8 ± 8.6°). Joint motion varied from room to room, even for a given task. For example, subjects walked fastest in the living room (1.0 ± 0.2 m/s) and slowest in the bathroom (0.78 ± 0.10 m/s), while the mean maximum ankle dorsiflexion in the living room was significantly higher than that in the bathroom (mean difference: 4.9°, *p* = 0.002, Cohen’s d = 1.25). This study highlights the dependency of both upper and lower limb joint motion during activities of daily living on the internal home environment. The digital twin modeling framework reported may be useful in planning home-based rehabilitation, remote monitoring, and for interior design and ergonomics.

## 1. Introduction

Mobility is broadly defined as the ability to move oneself within community environments. It is integral to active aging and intimately linked to health status and quality of life [1,2]. Human movement conditions, such as stroke, osteoarthritis, and Parkinson’s disease, affect approximately one in four people globally by impairing joint function [3]. This can significantly impact physical and mental health by lowering independence and restricting employment and community involvement [4]. Older adults with mobility conditions are particularly susceptible to injuries, especially within the home environment, where most unintentional falls and accidents occur [5]. The ability to measure and model human movement impairment is thought to be critical in preventative care, such as exercise therapy and rehabilitation.

Traditional approaches to assessing mobility impairment require repeated visits to a clinic, which is time-consuming and costly. Furthermore, standard mobility assessments such as clinical gait analysis are typically performed over a short time span in a controlled clinical setting. These conditions bear little resemblance to the real-world environment that one interacts with. The way a person moves in their own physical living space over time, interacting with objects such as doors, stairs, and furniture, is a vital determinate of their mobility and behavior [6]. The environment in which a person lives may facilitate active or sedentary behavior, present or mitigate risk of falls and injury, and ultimately affect a person’s psychological state and quality of life [7]. Measurement of motion in the home setting has the potential to encapsulate movement variability between living spaces and rare events that may not otherwise be captured in the lab setting [8]. Yet, 3D motion analysis of the whole body throughout the home setting is rarely performed, and the way in which people interact with the internal living environment remains poorly understood.

Human mobility has previously been quantified using markerless motion analysis cameras. However, occlusion of body movement, constraints on capture volume, and privacy concerns have limited their use beyond the laboratory setting [9,10]. In contrast, wireless, wearable devices can achieve unobtrusive motion joint analysis without restriction in capture volume. This includes optical fiber–based wearable sensors, which detect mechanical deformation and strain by measuring changes in transmitted or reflected light intensity or wavelength. These devices have shown high sensitivity to joint motion and muscle activity [11]. Smart photonic wearable sensors can also measure biomechanical signals, such as micro-strains, through resonance-wavelength shifts [12]. Among wearable technologies, inertial measurement units (IMUs) have been most widely used to directly quantify the relative angular motion of segments and joints [13]; however, none of these systems can delineate the absolute position of the body. Real-time indoor spatial tracking of human locomotion has been achieved using geo-location data from ultra-wideband (UWB) tracking systems [14,15], but this technology has yet to be employed for human mobility measurement in the home setting [16,17].

The aims of this exploratory pilot study were twofold. Firstly, to develop a digital twin of a fully furnished apartment that integrates real-time joint motion and spatial location tracking of subjects into a 3D digital reconstruction of the entire internal living environment; and secondly, to use this model to classify and evaluate joint motion during a diverse range of activities of daily living performed by healthy adults throughout the home setting. It was hypothesized that both lower limb joint motion during walking and upper limb joint motion during activities of daily living would vary significantly by room throughout the home setting, even for the same given task. The findings of this study will be useful for mobility monitoring and planning home-based rehabilitation protocols, and in developing fall prevention strategies, for example, in aged care facilities.

## 2. Materials and Methods

### 2.1. Motion Analysis Experiments

**Figure 1:**
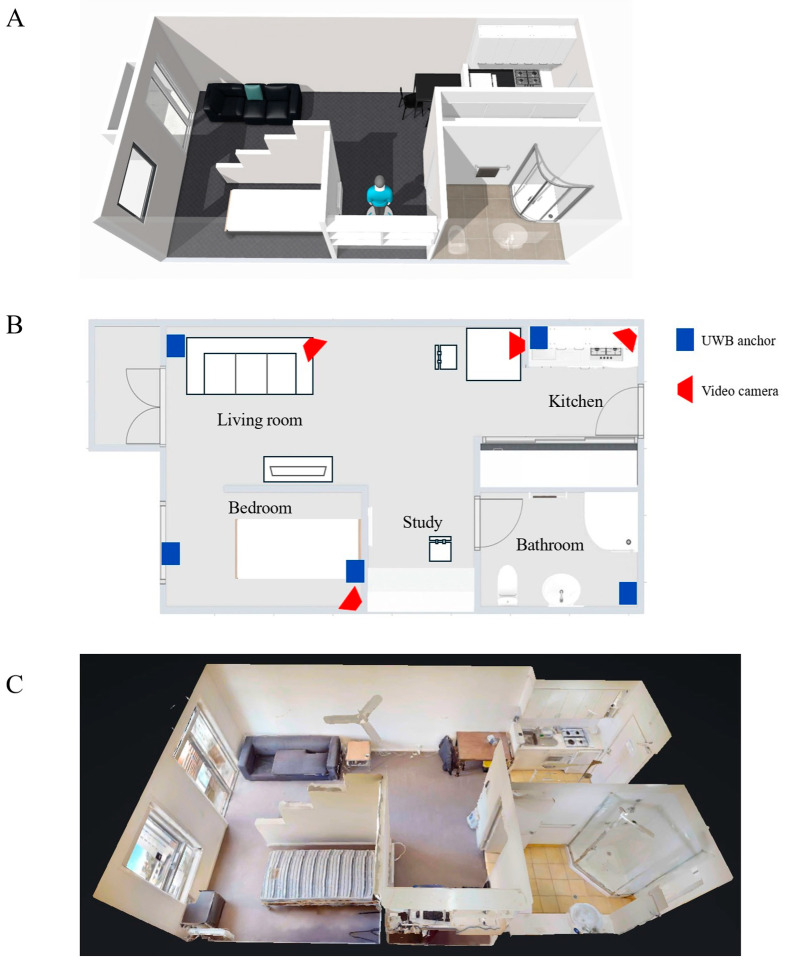
Schematic diagram illustrating layout of the single-bedroom apartment (**A**), its floor plan (**B**), and a 3D digital model of the apartment created using a Matterport camera (**C**). In subfigure (**B**), locations of ultra-wide band (UWB) anchors are shown as blue rectangles, and video cameras are shown as red trapezoids.

Ten healthy subjects (6 female and 4 male; age: 27.7 ± 1.8 years; height: 172.1 ± 7.8 cm; weight: 66.1 ± 6.3 kg) were recruited and provided written, informed consent. Human movement evaluation was conducted inside a fully furnished single-bedroom apartment comprising a kitchen, living room, study, bathroom, and bedroom (Figure 1A). This study had ethical approval (2023-25000-38049-6).

Each subject performed nineteen activities of daily living continuously throughout the living environment by using their preferred strategy and self-selected speed. Data collection lasted two hours for each participant (Table 1). Testing occurred in two phases, which included (1) operator-instructed activity sequences and (2) self-directed activities. For the operator-instructed activities, subjects were asked to perform 10 predefined task sets comprising 19 activities of daily living over 100 min (see Supplementary Material Table S1). Task sets were performed in random order, and each concluded with a rest break of approximately 30 s. For self-directed activities, participants were given 20 min to perform their preferred selection of activities of daily living at any location within the apartment using their self-selected timing and task sequence.

**Figure 2:**
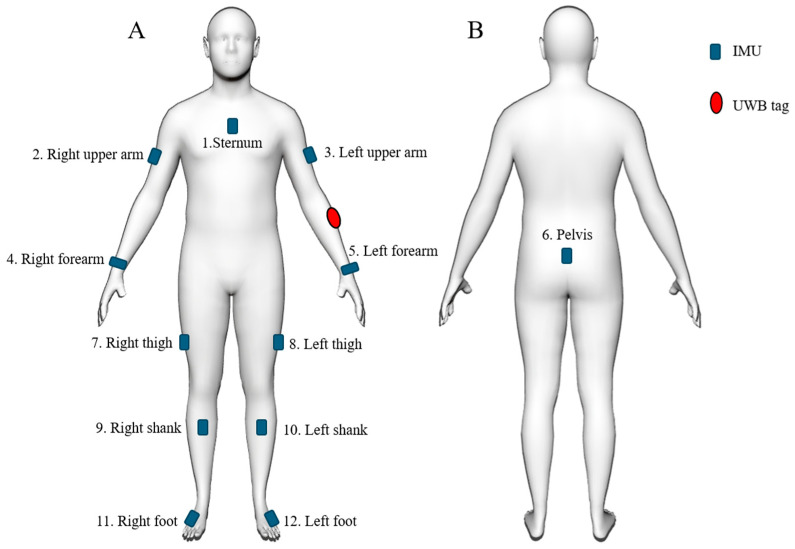
Illustration of approximate anatomical positions used for 12 IMUs (blue) and the UWB tag (red), including the anterior view (**A**) and the posterior view (**B**).

During testing, joint motion was simultaneously measured using 12 Blue Trident IMUs (Vicon, Oxford Metrics, Oxford, UK), which were self-placed on body segments by each subject, as described previously [18]. The IMU locations included the anterior middle thorax, posterior middle pelvis, left and right lateral upper arms, distal dorsal forearms, lateral middle thighs, proximal anterior shanks, and dorsal feet, with sensors placed in an arbitrary orientation (Figure 2). Each IMU comprised a triaxial accelerometer, gyroscope, and magnetometer sampling at 225 Hz. The three rotations of each IMU about its internal reference coordinate system were calculated using a commercial sensor fusion algorithm with drift correction (Invensense, TDK, San Jose, CA, USA). This calculation integrated raw acceleration, angular velocity, and magnetic heading data into a time-varying stream of quaternions. The relative orientation of each IMU with respect to the underlying anatomical segment, expressed as an orthogonal rotation matrix, was then calculated using averaged accelerometer data that were derived from two predefined static calibration poses undertaken before testing [19]. Three-dimensional joint angles of the shoulder (humerothoracic plane of elevation, elevation, and axial rotation), hip (flexion-extension, abduction–adduction, and axial rotation), knee (flexion–extension, abduction–adduction, and axial rotation), ankle (dorsi-plantar flexion, inversion–eversion, and axial rotation), and trunk inclination (plane of inclination, inclination, and axial rotation) were calculated in real-time during testing using an established Euler angle decomposition [18].

During testing, the spatial location of each participant in the home environment was simultaneously recorded using a UWB system, which comprised five UWB anchors (Pozyx, Belgium) and a UWB tag that was attached to each participant’s left forearm. The real-time location of the UWB tag was calculated by an onboard processor using a multilateration algorithm that processes the time difference of arrival (TDOA) of signals from the UWB tag to the anchors [20], with a sample rate of approximately 5 Hz and a tracking accuracy of within 30 cm [21]. The UWB anchors were mounted on the interior walls approximately 1.8 m above the floor using double-sided acrylic tape (Figure 1B). Both IMU and UWB data streams were resampled at 100 Hz and synchronized. The UWB data were used to calculate three parameters as follows: instantaneous overground walking speed, total distance traveled within the internal environment, and time spent in each room. Instantaneous speed was derived from the displacement–time ratio over 0.2 s intervals. The total distance was obtained by summing all instantaneous displacements across the recording period. The time spent in each room was the cumulative duration of the samples located within that room’s boundaries. Four video cameras (Google Nest Cam, Google, Mountain View, CA, USA) were placed in the lab space, with full visual coverage of the rooms (Figure 1B). These cameras were used to capture each subject’s movements during testing to support timestamping the beginning and end of each activity. Data sampling for the video cameras, UWB system, and IMUs were synchronized. 

### 2.2. Digital Twin Creation

**Figure 3:**
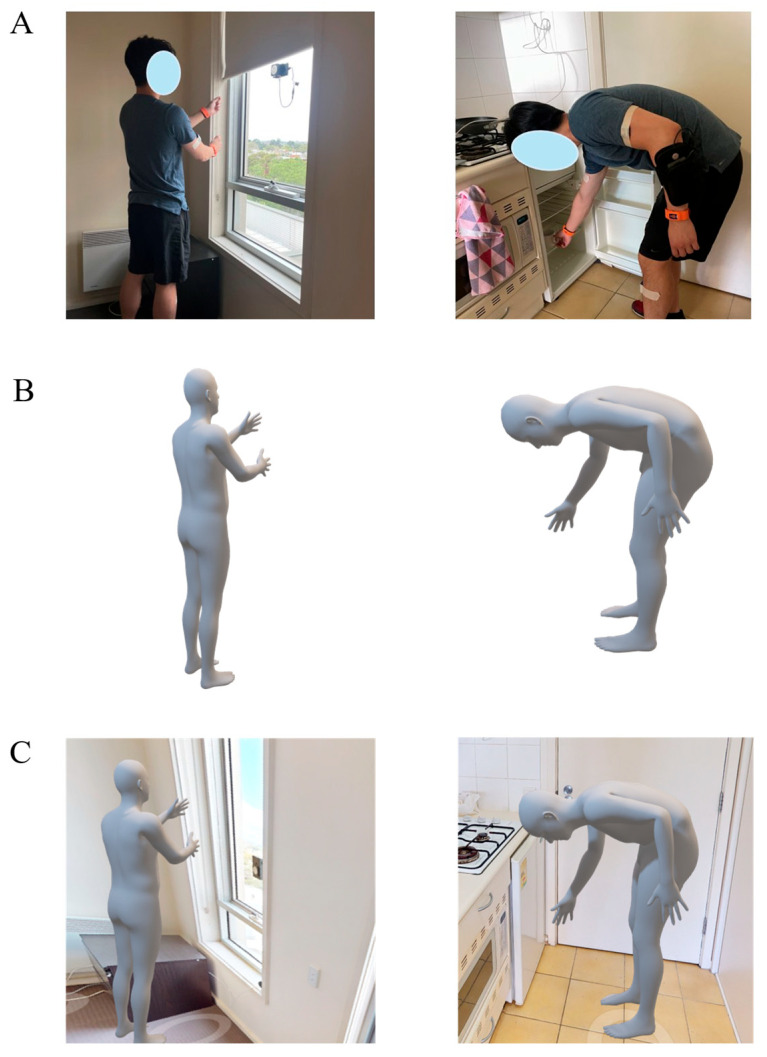
Illustration of digital twin model of the home setting and a participant performing activities of daily living, including a subject photographed while performing blind pulling in the bedroom and reaching to open a fridge door in the kitchen (**A**); the skinned multi-person linear model (SMPL) render of the participant performing the same pulling and reaching tasks (**B**); and integration of the SMPL into the digital reconstruction of the home setting (**C**). For further information about activities of daily living, see Table 1 and Supplementary Material Table S1).

A digital twin model of the furnished apartment was created by combining 3D imaging and digital reconstruction of all internal living spaces, together with real-time joint motion analysis and spatial location tracking of the subjects as they performed activities of daily living throughout this home setting. First, a 3D reconstruction of all interior walls, furniture, fittings, doors, and windows was performed using 3D photogrammetry (Figure 1C). This was achieved using a Matterport camera (Sunnyvale, USA) comprising high-resolution RGB cameras and depth sensors. Each room was scanned separately, and a 3D model was generated by aligning the collected images and depth measurements from multiple viewpoints. Second, the model’s coordinate system was registered to that of the UWB data so that the real-time location of each subject could be visualized during testing. Finally, a skinned multi-person linear model (SMPL)—a form of digital avatar—was created for each subject and placed within the digital model of the apartment in order to visualize human movement in real-time during testing. The SMPL poses were generated by actuating joint armatures using the measured joint angles from IMU data, while the spatial position of the avatar within the digital apartment was driven using the UWB data (Figure 3). Visualization of the digital twin was supported using Autodesk Maya (Alias Systems Corporation, Toronto, ON, Canada).

### 2.3. Activity Classification and Human Movement Assessment

K-Nearest Neighbor (KNN) and Support Vector Machine (SVM) with Radial Basis Function (RBF) kernel classifiers were used to classify the 19 predefined activities using IMU and UWB data. The time histories of joint angles calculated from the IMUs were segmented into windows with varying lengths, ranging from 0.1 to 2 s, with a 50% overlap. KNN and SVM kernels were trained using all window sizes. Additionally, KNN classifiers were trained using 5 to 99 neighbors. Key kinematic features were extracted from each window and used to train the model. All activities were manually labeled (segmented) from timestamped video footage and were associated with room name classes derived from UWB data. Activity classification accuracy was evaluated using a 10-fold subject-independent (leave-one-subject-out) cross-validation. This was achieved by training a model on data from 9 subjects and testing on the left-out subject, then repeating this process until each subject had been used as a test subject. A confusion matrix, created by concatenating all activity predictions from the 10 tests, was used to display individual activity classification accuracy over each task.

For each subject, the total duration of each activity and number of activity occurrences were delineated using the machine learning-predicted activity timestamps. The averaged maximum range of motion for the shoulder and lower limb joints was computed using the IMU data for each activity. At the shoulder, the maximum humerothoracic plane of elevation and elevation angles were calculated and used as measures of upper limb kinematics. The 0° reference for the humerothoracic plane of elevation was defined in the frontal plane, with 90°-shoulder plane of elevation corresponding to the anterior sagittal plane [22]. Similarly, for the lower limb, maximum hip flexion, maximum knee flexion, maximum ankle dorsiflexion, and maximum ankle plantarflexion were calculated for all activity occurrences.

### 2.4. Data Analysis

The automatic model-predicted accumulated activity duration, total number of activity occurrences, and averaged maximum range of motion for the shoulder and lower limb joints across all subjects were compared against those using manually labeled tasks. The labeled tasks served as the reference standard, with the start and end times identified from video data. Pairwise t-tests were then used to compare quantities between automatically recognized tasks and manually identified tasks. Additionally, the averaged maximum range of motion for the shoulder and lower limb joints between operator-directed and subject self-directed activities across all subjects was compared using pairwise t-tests. To account for multiple comparisons, False Discovery Rate adjustment was employed. The normality of the data was assessed and confirmed using the Kolmogorov–Smirnov test. A significance level of 5% was set for all comparisons, and standard deviation and 95% confidence intervals were used as a measure of data dispersion. Effect sizes were calculated using Cohen’s d [23].

## 3. Results

### 3.1. Spatial Motion Measurements

**Figure 4:**
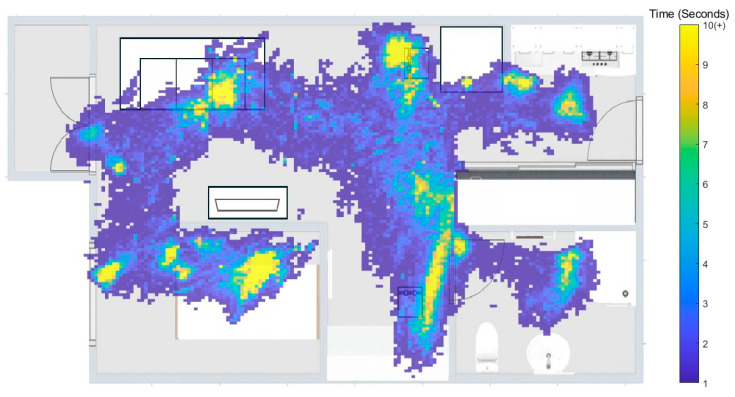
Heatmap of the average time participants spent in each living space.

Subjects spent an average 36.4 ± 5.6 min, 11.1 ± 5.6 min, 31.0 ± 5.1 min, 30.5 ± 5.6 min, and 17.5 ± 5.1 min in the bedroom, kitchen, living room, study, and bathroom, respectively (Figure 4). The locations where subjects spent the most time were on the bed in the bedroom (16.8 ± 2.3 min), at the table in the kitchen (8.4 ± 2.7 min), on the sofa in the living room (13.4 ± 2.0 min), at the desk in the study (12.3 ± 3.0 min), and in front of the mirror in the bathroom (9.9 ± 3.7 min). The average walking speed for all subjects was 0.79 ± 0.13 m/s in the bedroom, 0.98 ± 0.18 m/s in the kitchen, 1.00 ± 0.20 m/s in the living room, 0.94 ± 0.24 m/s in the study, and 0.78 ± 0.10 m/s in the bathroom.

### 3.2. Activity Classification

**Figure 5:**
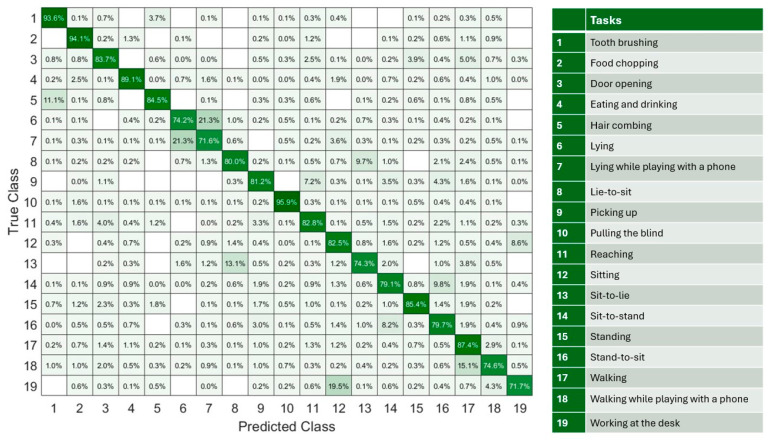
A confusion matrix for classifying 19 activities of daily living, based on concatenated results from 10 leave-one-subject-out tests. The color scale represents the percentage of classification counts, ranging from white (0%) to dark green (100%).

The highest mean classification accuracy during testing of all participants was achieved when using the KNN with nine neighbors (82.3%) (Table 2). The highest accuracy task classification occurred for blind pulling (95.9%), food chopping (94.1%), and tooth brushing (93.6%) (Figure 5). Six activities were misclassified by over 10%. This included 11.1% of hair-combing tasks that were misclassified as tooth brushing, 21.3% of lying down while playing with a phone was misclassified as lying down, 21.3% of lying down was misclassified as lying down while playing with a phone, 13.1% of sit-to-lie was misclassified as lie-to-sit, 15.1% of walking while playing with a phone was misclassified as walking, and 19.5% of working at the desk was misclassified as sitting. While the number of predicted task occurrences were not significantly different to the number of measured occurrences in most cases (*p* > 0.05), the classification model predicted a significantly higher number of lie-to-sit occurrences than what were measured (mean difference: 6.8, 95% CI: [4.0, 9.6], *p* = 0.019, Cohen’s d = 0.71) (Table 3).

### 3.3. Joint Motion During Activities of Daily Living

There were no significant differences in kinematics for the tasks performed in the operator-instructed phase compared with the same tasks performed in the self-directed activity phase (*p* > 0.05). For example, there were no significant differences in the maximum shoulder plane of elevation and elevation angle when subjects were instructed to perform door opening and reaching tasks, as compared to when subjects chose to perform these tasks at their own volition (*p* > 0.05) (Supplementary Material Table S2). No significant differences were observed in the model-predicted averaged maximum shoulder plane of elevation and elevation angles compared to the measured values (*p* > 0.05) (Table 4). Hair combing required the highest shoulder elevation of all upper limb tasks (124.2 ± 21.2°) while food chopping involved the lowest shoulder elevation (37.8 ± 9.8°). Blind pulling involved the largest maximum shoulder plane of elevation anterior to the frontal plane (112.0° ± 11.7°). Upper limb joint motion during the reaching-related tasks varied significantly depending on which room these tasks occurred in (*p* < 0.05). For example, the maximum shoulder elevation angle during reaching in the study (86.3 ± 17.3°) was significantly higher than that in the kitchen (mean difference: 19.5°, 95% CI: [2.2°, 36.9°], *p* = 0.032, Cohen’s d = 1.17).

No significant differences were observed in the maximum hip flexion, knee flexion, ankle plantarflexion, and dorsiflexion angles during sit-to-stand and walking between the measured operator-instructed tasks and measured self-directed activities (*p* > 0.05) (Supplementary Material Table S3). Sit-to-stand required the largest dynamic range of motion, with a predicted maximum hip flexion at 75.7 ± 10.3°, knee flexion at 91.8 ± 8.6°, and ankle dorsiflexion at 17.7 ± 5.9°, while sit-to-lie involved the greatest maximum ankle plantarflexion at 29.7 ± 10.1° (Table 5 and Table 6).

Lower limb joint motion during activities of daily living was strongly dependent on the room in which the specific task was undertaken. For example, the maximum knee flexion predicted during sit-to-stand in the bedroom (93.4 ± 9.3°) was significantly higher than that in the study (mean difference: 6.3°, 95%CI: [1.7°, 10.9°], *p* = 0.014, Cohen’s d = 0.85). During walking, the maximum ankle dorsiflexion in the living room (15.7 ± 3.4°) was significantly higher than that in the bathroom (mean difference: 4.9°, 95%CI: [2.5°, 7.3°], *p* = 0.002, Cohen’s d = 1.25), and the maximum ankle plantarflexion in the living room (18.3 ± 5.6°) was also significantly higher than that in the bathroom (mean difference: 5.9°, 95%CI: [1.4°, 10.3°], *p* = 0.016, Cohen’s d = 1.10).

## 4. Discussion

The objective of this study was to develop a digital twin model for the measurement, modeling, and visualization of human mobility in the home setting, and to use this to classify tasks and measure joint motion during activities of daily living that were performed over continuous data collection periods. In support of the study hypotheses, upper and lower limb joint motion was dependent on the home’s internal environment and how participants chose to interact with it, and this varied markedly between rooms. For example, significant differences were observed in gait speed and lower limb joint kinematics while walking in different rooms, as well as in upper limb joint angles for the same reaching tasks performed in different rooms.

In the present study, walking speed in the home setting for healthy adults averaged 0.9 m/s, while overground walking speed in the laboratory setting has been recorded between 1.3 m/s to 1.4 m/s [24,25,26]. This lower gait speed is likely due to the limited straight, unobstructed walking spaces and the presence of furniture (obstacles), which requires visual awareness and may increase attentional resources and cognitive demand. This finding aligns with that of Roth et al., who found that gait in the home setting is generally slower than that in controlled outdoor or laboratory environments [27]. We also observed that walking speed and lower limb joint motion varied within the home setting, with the lowest walking speeds in the bathroom (0.79 ± 0.13 m/s) and the highest in the living room (1.00 ± 0.20 m/s). Consequently, maximum ankle plantarflexion in the bathroom was 5.9° lower than that in the living room. These findings, which highlight marked variability of joint motion within the home setting, underscore the value of continuous remote monitoring for evaluating mobility over extended periods and may ultimately be useful in early detection of sedentary behavior or in assessing fall risk. The observed differences in joint motion between rooms suggest that interior design elements, including doorway width, furniture spacing, and fixture height, can influence movement strategies. For instance, confined spaces such as bathrooms induce more cautious gait patterns in older adults, characterized by slower walking speeds and reduced ankle range of motion [28]— a strategy that may ultimately reduce the risk of falling [28,29]. Future studies using our framework ought to explore the influence of obstacles, walking surfaces, and dual task conditions on walking patterns in the home setting.

The present study showed that habitual upper limb movements in the home setting produced a wide range of shoulder elevation, ranging from 37.8° (food chopping in the kitchen) to 124.2° (hair combing in the bathroom). In contrast, the humeral plane of elevation was generally invariant to the task, and the humerus remained close to the sagittal plane for most activities. The sagittal plane may be mechanically advantageous in upper limb elevation due to the recruitment of prime movers such as the anterior deltoid and clavicular sub-region of the pectoralis major in forward elevation. These findings indicate the clinical relevance of flexion in executing forward lifting and pushing tasks, suggesting the sagittal plane as a key target movement for shoulder muscle strengthening in rehabilitation and exercise-based therapy.

Different activities of daily living that shared similar movement features or poses resulted in higher classification errors. For instance, sagittal plane joint angles were not significantly different between lying down while playing with a phone and lying only, resulting in a 21.3% mislabeling rate between these activities. The reason for this mislabelling is that the machine learning model was unable to distinguish key features of phone use, which had a high dependence on small hand and wrist movements. This finding is similar to that of Leutheuser et al., who reported a percentage of confusion up to 46% between indoor ergometer cycling at two resistance levels using a hierarchical machine learning classifier due to undistinguishable joint motion patterns [30]. The use of feature selection techniques such as the Uniform Manifold Approximation and Projection (UMAP) method prior to model training could further enhance classification accuracy. These techniques reduce the feature space to the most relevant dimensions and improve the performance of KNN classifiers, which are more effective with lower-dimensional features [31]. Nonetheless, our 10-fold subject-independent model cross-validation accuracy of 82.3% across 19 activities of daily living was comparable to the 85.8% classification accuracy achieved by Leutheuser et al. for thirteen repetitive tasks [30]. Our classification accuracy was also robust to variants of tasks, for example, the ‘reaching’ class included opening a small, floor-mounted bar fridge, picking up a book from a high shelf, or grasping a cup from a table. However, the choice of KNN over SVM was based on marginally higher classification accuracy. Future studies ought to explore multi-modal data analytics and deep neural network approaches to consolidating data from different sources for more robust feature detection and task classification. This might include the use of heart rate and blood oxygen saturation monitors, skin conductance measurements, and eye tracking.

The digital twin presented in this study is an extension of recent research exploring smart-home digital twins for human biomechanics. A previous modeling framework was used to create a virtual home environment containing furniture that individuals in the home could interact with [32]. Other studies have introduced digital twins of human body segments for continuous measurement of joint kinematics and kinetics using wearable sensors [33,34]. Our digital twin performed reliably in both task recognition and joint motion measurement throughout the home environment. The mean classification accuracy of our framework (82.3%) exceeded that of a recently proposed framework that reconstructed a virtual apartment and classified human activities using synthetic ambient-sensor data such as binary motion, contact, and light sensors (81.6%). Moreover, we observed no significant differences between the predicted and measured maximum shoulder, hip, knee, and ankle flexion angles across diverse daily tasks. This result is comparable to the findings of Zhou et al., who developed an IMU-driven digital twin for human motion capture and reported errors below 6° in predicted knee flexion angles during static poses and walking [34].

A limitation of this study arose from the process of supervised learning, where a finite number of activity classes were predefined prior to model training [35]. This limitation presents a challenge in future applications where individuals may engage in diverse activities, undertake new tasks, and adapt or evolve movement patterns to changes in their living environment. Future research ought to explore open-set recognition frameworks in which a model is capable of detecting activities not part of model training [36], and continual learning approaches that incorporate new activity classes over time. The dataset employed in the present study was based on joint motion data obtained from 12 IMUs, a quantity impractical for daily life applications. Reducing the number of sensors required for activity recognition ought to be a focus of future research [37]. Finally, this was an exploratory pilot study of ten healthy participants, and the findings may not represent the broader population, including older adults or individuals with neuromuscular conditions. Nonetheless, the dataset comprised continuous and diverse activities of daily living over extended periods in a realistic setting, providing a sufficient basis for model-based activity recognition and mobility assessment.

## 5. Conclusions

In the present study, a novel digital twin modeling framework was developed to reconstruct and visualize the internal home environment of an apartment and monitor how subjects move and interact with this environment. The framework was capable of classifying 19 movement activities and their variants with an accuracy of 82.3%. Both upper and lower limb joint motion were strongly dictated by the internal environment and how participants chose to interact with it. This included gait speed and upper limb kinematics, which varied substantially throughout the home setting, even for the same task. The digital platform developed may support continuous real-time assessment of joint function in the home setting, with applications in telemedicine, rehabilitation, and remote monitoring for fall prevention, as well as in ergonomics and interior design.

## Figures and Tables

**Table 1:** Descriptions of activities of daily living employed in the present study.

Activities	Description
Tooth brushing	Standing in front of a mirror with the dominant hand holding a toothbrush and moving repetitively across the teeth in a back-and-forth motion
Food chopping	Standing at the food preparation bench in the kitchen, with one hand holding a vegetable securely while the other hand grips a knife to chop the food in a repetitive motion
Door opening	Grasping the bathroom or balcony door handle, twisting to unlock it, then pushing or pulling to open, and then taking one or more steps to enter
Eating and drinking	Sitting at the kitchen table on a chair with food items, such as biscuits, fruit, or pasta, and a drink in a cup placed on the table. The subject consumes food using their selected strategy
Hair combing	Standing in front of the mirror with one hand holding a comb and moving it repetitively through the hair while the other hand holds the hair in place if necessary
Lying	Lying on the bedroom bed with the body in a horizontal position, facing up
Lying while using a phone	Lying on the bedroom bed with the body in a horizontal position, facing up, with one or both hands holding and operating a smartphone
Lie-to-sit	Transitioning from lying supine on the bed to a seated position with both feet flat on the floor
Picking up an object	Starting from a standing position, lowering the body by bending at the back or lower limbs, grasping an object on the ground, and then returning to the standing height
Pulling the blind	Standing beside the window in the bedroom and repetitively pulling the roller chain of the blind to raise or lower it
Reaching	Reaching to grasp and pick up an object positioned at either head-, waist-, or knee-level. Reaching may include opening a floor-level bar fridge, picking up an object from the fridge, or grasping a book from a high shelf
Sitting	A seated posture on any seat-level surface, including a bed, sofa, kitchen chair, or computer chair, with both feet flat on the floor and the torso upright
Sit-to-lie	Transitioning from a seated position on the bed to a lying position
Sit-to-stand	Movement of the body from a seated position on a seating surface and both feet flat on the floor to an upright standing position
Standing	An upright posture with the subject standing on both feet and their arms to the sides
Stand-to-sit	Movement of the body from an upright standing position to a seated position on any seating surface with both feet flat on the floor
Walking	Walking continuously, over 3 or more steps at the subject’s preferred speed
Walking while using a phone	Walking while operating a smartphone with one or both hands
Working at a desk	Sitting at a desk on a study chair and writing on a piece of paper, or typing on a computer keyboard

**Table 2:** Activity classification accuracy was evaluated based on 10-fold cross-validation using different machine learning models and window sizes.

Window Size (s)	Mean Cross-Validation Accuracy (%)				
	KNN5	KNN9	KNN49	KNN99	SVM Kernel
0.1	76.2	77.0	77.9	77.6	74.1
0.2	78.2	78.6	78.7	78.0	76.3
0.3	79.5	79.8	79.4	78.5	77.2
0.4	80.4	80.6	79.5	78.6	77.8
0.5	81.0	81.1	80.1	78.9	77.8
0.6	81.4	81.5	80.5	79.9	78.7
0.7	81.5	81.6	80.8	80.4	79.1
0.8	81.6	81.8	80.9	80.6	79.3
0.9	81.6	81.6	81.0	80.5	79.5
1.0	82.0	81.8	81.2	80.6	79.9
1.1	81.9	82.1	81.5	80.3	79.8
1.2	81.7	81.9	81.6	80.2	79.9
1.3	82.1	82.3	82.0	80.7	80.6
1.4	81.8	82.2	82.1	80.7	80.3
1.5	81.7	82.1	82.0	80.7	79.9
1.6	81.6	82.0	82.3	80.7	80.4
1.7	81.8	82.0	82.3	80.7	80.5
1.8	82.1	82.1	82.2	80.7	79.7
1.9	82.3	82.4	82.3	80.7	79.9
2.0	82.1	82.2	82.1	80.9	80.1

The color scale represents the magnitude of classification accuracy, ranging from dark red (lowest) to dark blue (highest). The models included KNN with 5, 9, 49, and 99 neighbors, and Kernel SVM. Time-series data, including IMU joint angles, room name, and the activity labels, were automatically segmented into windows with sizes ranging from 0.1 to 2.0 s, with increments of 0.1 s. The activity label and room name that took up the highest proportion of each window were used to assign the activity label and room name of that window. Key kinematic signal features were calculated from IMU-based joint-angle data within each data window, including the mean and standard deviation of the joint angles, as well as those of their first and second derivatives. These features were selected to represent posture, movement speed, and sudden changes in motion. A 50% overlap was applied between consecutive windows. A KNN model with 9 neighbors and a 1.3 s window was selected for joint mobility analysis, as it resulted in the highest overall accuracy while being of sufficiently short duration to support classification of all movements.

**Table 3:** Mean and standard deviation of the total number of activity of daily living occurrences, and the accumulated (total) duration of each activity for each participant when tasks were manually segmented using the video camera data or automatically predicted using the machine learning model. Differences between measured and predicted data were calculated using paired *t*-tests, and the resulting *p*-values were given.

Activity		Number of Occurrences				Accumulated Duration		
		Measured	Predicted	*p*-Value		Measured	Predicted	*p*-Value
Tooth brushing		6.9 ± 2.3	7.0 ± 2.5	0.795		123.7 ± 28.6	130.3 ± 29.6	0.374
Food chopping		6.7 ± 2.0	7.1 ± 2.7	0.589		145.8 ± 40.0	150.9 ± 45.8	0.491
Door opening		35.6 ± 4.8	33.8 ± 3.5	0.245		162.1 ± 19.4	155.1 ± 14.4	0.374
Eating and drinking		6.7 ± 1.7	6.9 ± 2.3	0.795		126.8 ± 36.8	125.7 ± 54.7	0.939
Hair combing		7.0 ± 2.1	7.4 ± 3.2	0.590		111.4 ± 44.2	112.5 ± 46.0	0.900
Lying		6.1 ± 1.3	6.6 ± 2.8	0.795		91.3 ± 15.2	102.0 ± 16.0	0.374
Lying while using phone		5.6 ± 1.9	6.4 ± 2.8	0.431		105.3 ± 47.0	114.8 ± 56.9	0.715
Lie-to-sit		43.9 ± 8.5	50.7 ± 10.4	0.019		127.0 ± 34.1	131.1 ± 32.1	0.632
Picking up an object		37.0 ± 4.5	42.7 ± 7.6	0.109		168.0 ± 24.1	162.3 ± 32.0	0.608
Pulling the blind		4.8 ± 1.4	4.4 ± 1.5	0.171		114.1 ± 51.8	118.3 ± 54.3	0.632
Reaching		36.3 ± 5.3	43.4 ± 10.0	0.070		171.3 ± 18.3	175.6 ± 22.0	0.374
Sitting		18.2 ± 4.5	22.0 ± 9.2	0.070		173.3 ± 57.0	185.2 ± 66.8	0.429
Sit-to-lie		44.8 ± 7.6	40.4 ± 9.8	0.048		132.7 ± 28.5	118.9 ± 24.6	0.361
Sit-to-stand		86.6 ± 18.5	96.9 ± 23.0	0.099		149.4 ± 43.5	152.5 ± 36.8	0.737
Standing		14.3 ± 5.5	14.8 ± 5.1	0.590		100.5 ± 43.3	96.5 ± 45.2	0.374
Stand-to-sit		90.4 ± 17.1	100.2 ± 22.0	0.108		158.3 ± 34.6	165.7 ± 39.5	0.492
Walking		25.4 ± 6.3	29.4 ± 8.4	0.133		168.0 ± 38.4	164.2 ± 42.3	0.374
Walking while using phone		15.0 ± 2.9	17.1 ± 5.1	0.371		126.5 ± 23.8	121.5 ± 23.7	0.374
Working at desk		7.2 ± 2.0	7.1 ± 3.3	0.912		169.3 ± 76.0	157.5 ± 74.1	0.374

**Table 4:** Mean and standard deviation of the averaged maximum shoulder plane of elevation and plane of elevation for each upper limb activity of daily living when tasks were manually segmented using the video camera data or automatically predicted using the machine learning model. Differences between measured and predicted data were calculated using paired *t*-tests, and the resulting *p*-values were given.

Activity		Max Shoulder Plane of Elevation (Degrees)				Max Shoulder Elevation (Degrees)		
		Measured	Predicted	*p*-Value		Measured	Predicted	*p*-Value
Tooth brushing		92.0 ± 10.1	89.5 ± 11.7	0.216		94.5 ± 15.7	93.4 ± 14.7	0.721
Food chopping		101.6 ± 30.6	102.4 ± 28.3	0.737		42.2 ± 10.6	37.8 ± 9.8	0.352
Door opening		83.4 ± 10.0	79.4 ± 12.9	0.645		76.9 ± 18.9	69.8 ± 10.6	0.352
Eating and drinking		97.8 ± 23.1	91.7 ± 23.8	0.216		70.8 ± 23.8	66.9 ± 23.8	0.352
Hair combing		94.7 ± 9.9	93.3 ± 10.2	0.216		126.7 ± 19.4	124.2 ± 21.2	0.352
Picking up an object		97.3 ± 18.1	93.0 ± 19.2	0.216		90.1 ± 14.7	86.7 ± 9.1	0.477
Pulling the blind		110.9 ± 9.0	112.0 ± 11.7	0.723		102.7 ± 19.2	101.3 ± 20.1	0.352
Reaching		94.2 ± 16.9	89.3 ± 10.8	0.216		72.4 ± 16.2	72.7 ± 9.4	0.937
Working at desk		85.3 ± 13.3	83.2 ± 19.2	0.737		76.5 ± 18.9	68.0 ± 22.3	0.352

**Table 5:** Mean and standard deviation of average maximum hip flexion and maximum knee flexion during each lower limb activity of daily living when tasks were manually segmented using the video camera data or automatically predicted using the machine learning model. Differences between measured and predicted data were calculated using paired *t*-tests, and the resulting *p*-values were given.

Activity		Max Hip Flexion (Degrees)				Max Knee Flexion (Degrees)		
		Measured	Predicted	*p*-Value		Measured	Predicted	*p*-Value
Lying		1.2 ± 8.3	−0.2 ± 9.6	0.323		1.5 ± 6.5	2.2 ± 5.4	0.690
Lying while using phone		0.0 ± 9.1	0.2 ± 9.8	0.900		2.3 ± 7.2	1.9 ± 8.3	0.738
Lie-to-sit		68.5 ± 15.5	63.5 ± 17.6	0.110		72.7 ± 7.5	71.2 ± 7.4	0.660
Sitting		70.6 ± 16.0	70.5 ± 14.9	0.900		80.6 ± 6.3	78.5 ± 6.8	0.300
Sit-to-lie		75.6 ± 9.9	72.1 ± 11.1	0.323		93.2 ± 9.3	92.0 ± 10.5	0.660
Sit-to-stand		75.8 ± 9.6	75.7 ± 10.3	0.900		91.9 ± 8.2	91.8 ± 8.6	0.903
Standing		3.8 ± 4.8	4.0 ± 4.1	0.900		5.6 ± 2.8	6.0 ± 3.0	0.690
Stand-to-sit		73.5 ± 8.9	72.9 ± 7.4	0.630		82.5 ± 12.6	80.0 ± 13.0	0.440
Walking		28.8 ± 5.3	27.6 ± 5.1	0.323		60.1 ± 5.5	58.4 ± 7.0	0.660
Walking while using a phone		25.2 ± 6.6	24.8 ± 6.2	0.900		59.9 ± 6.3	59.4 ± 6.9	0.738

**Table 6:** Mean and standard deviation of average maximum ankle dorsiflexion and maximum ankle plantar flexion during each lower limb activity of daily living when tasks were manually segmented using the video camera data or automatically predicted using the machine learning model. Differences between measured and predicted data were calculated using paired *t*-tests, and the resulting *p*-values were given.

Activity		Max Ankle Dorsiflexion (Degrees)				Max Ankle Plantar Flexion (Degrees)		
		Measured	Predicted	*p*-Value		Measured	Predicted	*p*-Value
Lying		4.3 ± 10.3	1.3 ± 13.1	0.402		16.7 ± 10.7	14.7 ± 13.8	0.630
Lying while using phone		0.7 ± 8.3	−2.0 ± 7.0	0.541		18.5 ± 18.2	18.9 ± 14.2	0.950
Lie-to-sit		5.1 ± 11.8	3.9 ± 10.9	0.466		30.2 ± 11.3	29.3 ± 11.2	0.268
Sitting		4.6 ± 9.1	4.1 ± 7.8	0.561		31.8 ± 8.8	30.7 ± 10.1	0.268
Sit-to-lie		11.8 ± 6.9	10.4 ± 8.5	0.402		10.6 ± 10.4	7.2 ± 10.4	0.268
Sit-to-stand		18.3 ± 5.6	17.7 ± 5.9	0.402		−0.7 ± 3.9	−0.8 ± 4.5	0.890
Standing		4.3 ± 3.4	4.3 ± 3.8	0.922		1.3 ± 3.9	0.9 ± 4.0	0.268
Stand-to-sit		17.8 ± 5.5	17.4 ± 5.4	0.402		0.1 ± 3.2	0.6 ± 2.9	0.268
Walking		13.4 ± 3.5	13.7 ± 3.3	0.466		18.0 ± 2.6	16.3 ± 2.5	0.268
Walking while using a phone		14.0 ± 4.3	11.7 ± 7.3	0.402		15.4 ± 4.0	15.3 ± 5.0	0.950

## Data Availability

The dataset is available on request from the authors.

## Supplementary Materials

The following supporting information can be downloaded at: https://www.mdpi.com/article/10.3390/s25247409/s1. Table S1. Predefined activity sequences for operator-instructed data collection. Table S2. Mean and standard deviation of the averaged maximum shoulder plane of elevation and shoulder elevation measured using the video camera data during operator-instructed and self-directed upper limb activities of daily living. Differences between operator-instructed and self-directed data were calculated using paired *t*-tests, and the resulting *p*-values given. Acronyms included: OI, operator instructed; and SD, self-directed. Table S3. Mean and standard deviation of the averaged maximum hip flexion, maximum knee flexion, maximum ankle dorsiflexion, and ankle plantar flexion measured using the video camera data during operator-instructed tasks and self-directed lower limb activities of daily living. Differences between measured and predicted data were calculated using paired *t*-tests, and the resulting *p*-values given. Acronyms included: OI, operator instructed; and SD, self-directed.

## Abbreviations

The following abbreviations are used in this manuscript:

IMU	Inertial Measurement Unit
UWB	Ultra-wide band
SMPL	skinned multi-person linear model
KNN	K-Nearest Neighbor
SVM	Support Vector Machine
