# Supplementary material for: Joint Function and Movement Variability During Daily Living Activities Performed Throughout the Home Setting: A Digital Twin Modeling Study
Source: Sensors (Basel). 2025 Dec 5;25(24):7409. doi: 10.3390/s25247409 (PMC12736529; doi:10.3390/s25247409)
Supplement: Supplementary file 1 [file sensors-25-07409-s001.zip › sensors-3975245-supplementary.pdf]

sensors

MDPI

Article

Joint Function and Movement Variability During Daily Living Activities Performed Throughout the Home Setting: A Digital Twin Modeling Study

Zhou Fang [1], Mohammad Yavari [1], Yiqun Chen [2], Davood Shojaei [2], Peter Vee Sin Lee [1], Abbas Rajabifard [2] and David Ackland [1,*]

[1] Department of Biomedical Engineering, University of Melbourne, Melbourne, VIC 3010, Australia; mohammad.yavari@student.unimelb.edu.au (M.Y.); pvlee@unimelb.edu.au (P.V.S.L.)
[2] Department of Infrastructure Engineering, University of Melbourne, Melbourne, VIC 3010, Australia
* Correspondence: dackland@unimelb.edu.au

Highlights

What are the main findings?

- A digital twin was developed to support real-time mobility and joint motion measurement and monitoring in the home setting.
- Activities of daily living, and the way they are executed, vary throughout the home setting, even for the same task.

What are the implications of the main findings?

- The findings have implications for home interior design and layout to improve mobility and reduce the risk of falls.
- The framework presented may be useful for real-time monitoring of movement in the home setting, as well as telemedicine and telerehabilitation.

Table S1. Predefined activity sequences for operator-instructed data collection.

Location	Task	Location	Task
Kitchen		Bedroom to kitchen	Walking
			Walking while playing with a phone
	Walking		Stand-to-sit on bed
	Walking while working with a phone		Sit-to-lie
	Reaching (opening the fridge)		Lying
	Reaching to a cup		Lying while playing with a phone
	Reaching (closing the fridge)		Sit-to-lie
	Food chopping		Lie-to-sit
	Stand-to-sit on chair		Sitting
	Eating and drinking		Sit-to-stand
	Sit-to-stand		Walking
	Standing		Reaching (opening the fridge)
	Reaching to a cup		Reaching to a cup

	Reaching (opening the fridge)<br>Reaching (returning the cup)		Reaching (closing the fridge)<br>Food chopping<br>Eating and drinking<br>Standing
Bedroom	Walking<br>Walking while playing with a phone<br>Pulling the blind<br>Walking<br>Standing<br>Stand-to-sit on bed<br>Sit-to-lie on bed<br>Lying<br>Lying while playing with a phone<br>Lie-to-sit<br>Sitting<br>Sit-to-stand	Living room to bedroom	Walking<br>Walking while playing with a phone<br>Stand-to-sit<br>Sitting on sofa<br>Sit-to-stand<br>Picking up an item<br>Pulling the blind<br>Stand-to-sit<br>Sit-to-lie on bed<br>Lying while playing with phone<br>Lying
Bathroom	Door opening<br>Walking<br>Walking while playing with a phone<br>Standing<br>Tooth brushing<br>Hair combing<br>Sitting<br>Door closing	Bedroom to bathroom	Walking<br>Walking while playing with a phone<br>Stand-to-sit on bed<br>Sit-to-lie<br>Lying<br>Lie-to-sit<br>Sit-to-stand<br>Pulling the blind<br>Walking to the bathroom<br>Door opening<br>Tooth Brushing<br>Hair Combing<br>Walking<br>Door closing
Study	Walking<br>Walking while playing with a phone<br>Walking<br>Picking up an item<br>Reaching to an item on the shelf<br>Stand-to-sit<br>Sitting<br>Working at the desk<br>Sit-to-stand<br>Standing	Study to kitchen	Walking<br>Walking while playing with a phone<br>Stand-to-sit<br>Sitting<br>Working at the desk<br>Sit-to-stand<br>Walking to the kitchen<br>Reaching (opening the fridge)<br>Reaching to a cup<br>Reaching (closing the fridge)<br>Food chopping<br>Stand-to-sit<br>Eating and drinking<br>Sit-to-stand

Living room	Walking<br>Walking while playing with a phone<br>Picking up an item<br>Standing<br>Stand-to-sit<br>Sitting on sofa<br>Sit-to-stand<br>Opening balcony door<br>Closing balcony door	Study to living room	Walking<br>Walking while playing with a phone<br>Reaching to an item on the shelf<br>Stand-to-sit<br>Sitting<br>Working at the desk<br>Sit-to-stand<br>Reaching to an item on the shelf<br>Picking up an item<br>Stand-to-sit on the sofa<br>Sitting

Table S2. Mean and standard deviation of the averaged maximum shoulder plane of elevation and shoulder elevation measured using the video camera data during operator-instructed and self-directed upper limb activities of daily living. Differences between operator-instructed and self-directed data were calculated using paired t-tests, and the resulting p-values given. Acronyms included: OI, operator instructed; and SD, self-directed.

**Task**	**Max shoulder plane of elevation (degrees)**			**Max shoulder elevation (degrees)**		
	**OI**	**SD**	**P-value**	**OI**	**SD**	**P-value**
Door opening	83.2 ± 12.4	83.4 ± 10.6	0.828	76.6 ± 24.9	79.5 ± 17.7	0.645
Reaching	95.8 ± 16.0	91.1 ± 11.3	0.226	77.2 ± 16.9	70.6 ± 23.2	0.366

Table S3. Mean and standard deviation of the averaged maximum hip flexion, maximum knee flexion, maximum ankle dorsiflexion, and ankle plantar flexion measured using the video camera data during operator-instructed tasks and self-directed lower limb activities of daily living. Differences between measured and predicted data were calculated using paired t-tests, and the resulting p-values given. Acronyms included: OI, operator instructed; and SD, self-directed.

**Task**	**Max hip flexion (degrees)**			**Max knee flexion (degrees)**			**Max ankle dorsiflexion (degrees)**			**Max ankle plantarflexion (degrees)**		
	**OI**	**SD**	**P-value**	**OI**	**SD**	**P-value**	**OI**	**SD**	**P-value**	**OI**	**SD**	**P-value**
Sit-to-stand	74.3 ± 7.5	77.0 ± 4.4	0.144	90.7 ± 11.6	93.7 ± 13.5	0.133	16.2 ± 9.0	19.8 ± 10.8	0.060	0.4 ± 7.2	-1.7 ± 8.8	0.642
Walking	29.2 ± 3.8	28.3 ± 4.0	0.310	59.8 ± 4.0	61.1 ± 6.3	0.744	17.6 ± 2.7	16.7 ± 6.8	0.596	21.9 ± 4.3	22.6 ± 5.2	0.720
